# Swinepox Virus Outbreak, Brazil, 2011

**DOI:** 10.3201/eid1710.110549

**Published:** 2011-10

**Authors:** Maria Luiza G. Medaglia, Adriana de Cássia Pereira, Tânia R.P. Freitas, Clarissa R. Damaso

**Affiliations:** Universidade Federal do Rio de Janeiro, Rio de Janeiro, Brazil (M.L.G. Medaglia, C.R. Damaso);; Centro de Patologia Animal, Campinas, Brazil (A.C. Pereira);; Laboratório Nacional Agropecuário de Minas Gerais–Ministério da Agricultura, Pecuária e Abastecimento, Pedro Leopoldo, Brazil (T.R.P. Freitas)

**Keywords:** Swinepox virus, vaccinia virus, poxvirus, swine, viruses, Brazil, letter

**To the Editor:** *Swinepox virus* (SWPV), which replicates only in swine, belongs to the *Suipoxvirus* genus of the *Poxviridae* family. It is the etiologic agent of a skin disease of pigs, characterized by generalized pustular lesions and associated with high rates of illness (occasionally >80%). It occurs mainly on farms with poor management and housing conditions and affects primarily pigs <3 months of age; adult pigs show milder signs. The disease is mechanically transmitted by pig lice or through direct animal contact (*1*). *Vaccinia virus* (VACV; *Orthopoxvirus* genus) also causes a similar pustular disease in pigs that is difficult to distinguish clinically from SWPV infections. VACV infections were common during smallpox vaccination campaigns, when VACV was transmitted to domestic animals from lesions of vaccinees (*1**,**2*).

Swinepox disease has a worldwide distribution, and 4 outbreaks of similar infections were reported in pig herds in Brazil during 1976–2001 (*3*). Nevertheless, the etiologic agents of these outbreaks have never been identified through molecular techniques. Specific virus identification in such infections is particularly relevant in Brazil, considering the persistence of VACV in nature in this country, causing frequent outbreaks of pustular skin disease in dairy cattle (*4**–**6*). Therefore, distinguishing between SWPV and VACV infections during outbreaks of pustular disease in pigs is essential for evaluating whether VACV infection might have spread to pigs and whether SWPV could be detected in Brazil.

We describe the molecular identification of SWPV as the etiologic agent of an outbreak of pustular disorder in pig herds. In November 2010 and January 2011, ≈850 of 3,460 animals on 3 pig farms in Holambra, São Paulo, Brazil, had generalized pustular lesions on the body, fever (38.0°C–39.7°C) and mild weight loss. Lesions evolved from macules or papules to umbilicated lesions with pustular content, followed by crusting (Figure A1). Secondary dermatitis was also noticed. Healing occurred after 3–4 weeks, but the disease started subsequently in previously healthy animals. Although the first clinical signs of disease started in the nursery units (pigs 40–50 days old), nearly 70% of the sick pigs were at the finishing units (pigs 127–134 days old), where elevated animal density and deficient sanitation conditions were observed. These findings may account for the high attack rate (nearly 50%) in finisher pigs, although overall illness was moderate (nearly 25%) when animals from all units were analyzed together. No deaths were associated with the outbreak, in concordance with the low death rates reported for SWPV infections (<5%) (*1*). The affected farms belonged to the same owner, who reported frequent movement of animals between the farms.

Scabs from 7 animals were used for DNA extraction (*4*), followed by PCR detection of poxvirus DNA (*7*). We used primers designed to anneal to gene regions conserved in different poxviruses: FP-A2L, 5′-TAGTTTCAGAACAAGGATATG-3′ and RP-A2L, 5′-TTCCCATATTAATTGATTACT-3′ directed the amplification of a 482-bp fragment of the virus late transcription factor–3 (www.poxvirus.org); primer sets for the DNA polymerase gene (543-bp fragment) and DNA topoisomerase gene (344-bp fragment) were previously described (*7*). Amplicons were directly sequenced as described (*4**,**5*). Consensus primers that specifically detect the full-length hemagglutinin gene of Eurasian-African orthopoxviruses were used to investigate VACV in the samples (*4*).

The nucleotide sequences obtained for the fragments of the DNA polymerase, DNA topoisomerase, and virus late transcription factor–3 of the clinical specimens were aligned with sequences from other poxviruses available in the public database (GenBank). They showed 100% nt identity with their orthologs of SWPV Nebraska strain. Concatenated amino acid alignments were used for phylogenetic inference (Figure). The clinical isolates and SWPV branched together in the phylogenetic tree with high bootstrap support. No amplification of the hemagglutinin gene was obtained, demonstrating that the animals were not infected with VACV. Samples were also negative for *Erysipelothrix* spp. (by PCR and ELISA) and porcine circovirus-2 (by PCR).

Outbreaks of swinepox disorders have been frequently reported in Europe, North America, and Oceania, and special attention has been given to congenital cases, which usually lead to high case-fatality rates (*2**,**8**,**9*). Our data identified SWPV as the cause of a recent outbreak in Brazil and suggest that previous outbreaks in the neighboring municipality of Campinas in 1976 and 1980 (*3*) may have been caused by SWPV as well because pigs are the only host and reservoir of the virus. Further sequencing analysis of the virus isolates will be necessary to characterize the strain of SWPV circulating in Brazil.

Recently, an outbreak of VACV-related disease in horses was reported in southern Brazil, which alerted the scientific community to the possible spread of this disorder to animal hosts other than dairy cattle (*10*). However, our data clearly demonstrate that this outbreak in pigs does not represent a spread of VACV infection, despite frequent reports of VACV-related outbreaks in dairy cows in São Paulo State (*6*). Therefore, the differential diagnosis of skin diseases of pigs might be a useful tool in epidemiologic surveys to access VACV spread and host range in Brazil.

**Figure:**
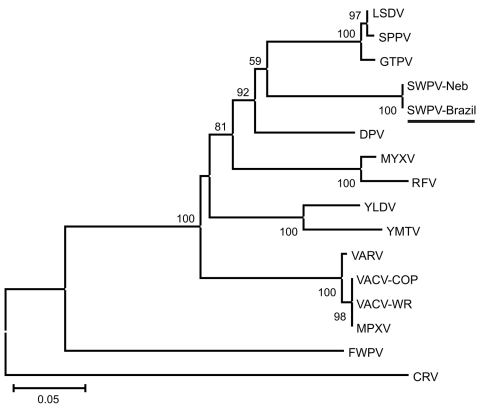
Phylogenetic tree based on the predicted amino acid sequences of fragments of the DNA polymerase, DNA topoisomerase, and viral late transcription factor-3 of the clinical isolate (GenBank accession nos. JF770341, JF770342, and JF770343) and 15 poxviruses. Sequences were aligned by ClustalX version 1.81 (www.clustal.org), and the concatenated alignments were used for phylogeny inference (MEGA4; www.megasoftware.net) opting for the neighbor-joining method and Poisson correction. We computed 1,500 replicates for bootstrap support. Values >50% are shown. The tree is drawn to scale, with branch lengths in the same units as those of the evolutionary distances used to infer the phylogenetic tree. Virus species and GenBank accession numbers: LSDV (lumpy skin disease virus; AF409137), SSPV (sheeppox virus; AY077834), GTPV (goatpox virus; AY077834), SWPV-Neb (swinepox virus Nebraska strain; NC_003389), DPV (deerpox virus; AY689437), MYXV (myxoma virus; NC_001132), RFV (rabbit fibroma virus; NC_001266), YLDV (Yaba-like disease virus; NC_002642), YMTV (Yaba monkey tumor virus; NC_002642), VARV (variola virus; NC_002642), VACV-COP (vaccinia virus Copenhagen strain; M35027), VACV-WR (vaccinia virus WR strain; NC_006998), MPXV (monkeypox virus; DQ011154), FWPV (fowlpox virus; NC_002188), CRV (crocodilepox virus; NC_008030). Virus isolated in this study is underlined.

## Figures and Tables

**Figure A1:**
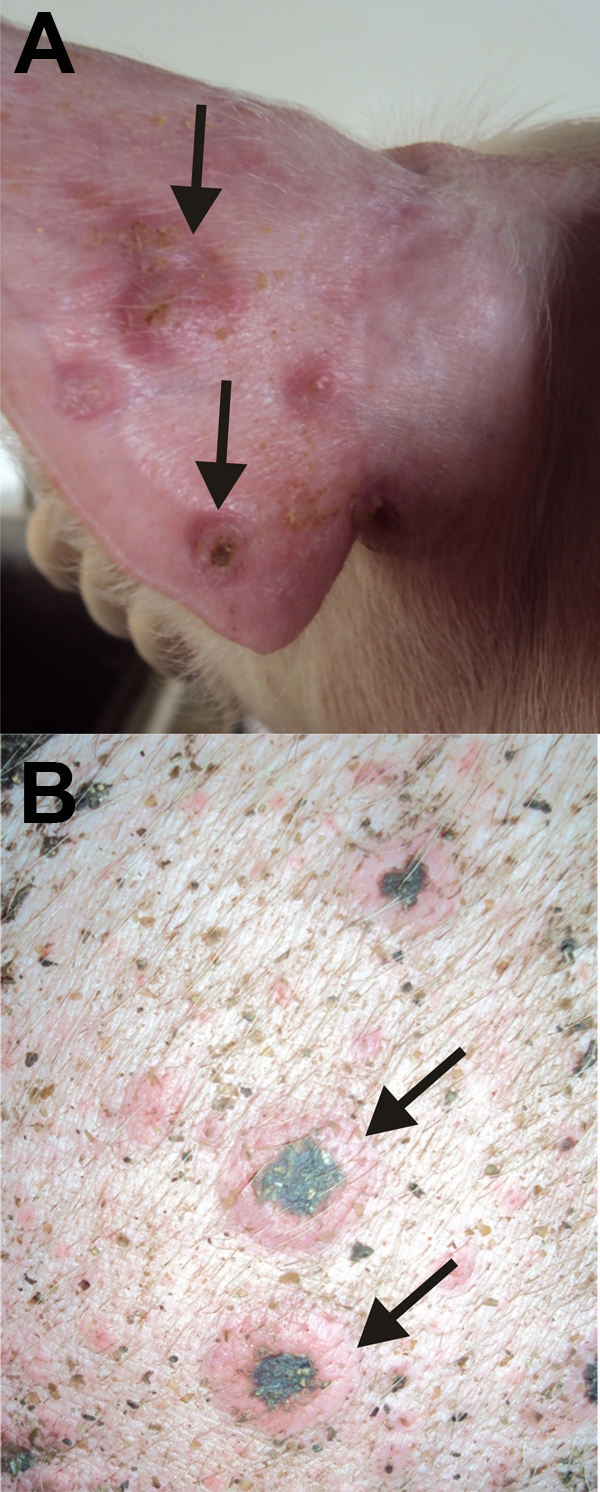
Lesions caused by swinepox virus, Brazil, 2011. A) Ear of a piglet in the nursery unit. Arrows indicate vesicles filled with pustular fluid. B) Trunk of a pig in the finishing unit. Arrows indicate umbilicated red lesions with black scabs in the center.
